# Path Planning for Mobile-Anchor Based Wireless Sensor Networks Localization: Obstacle-Presence Schemes

**DOI:** 10.3390/s21113697

**Published:** 2021-05-26

**Authors:** Dogan Yildiz, Serap Karagol

**Affiliations:** Electrical & Electronics Engineering Department, Ondokuz Mayis University, 55270 Samsun, Turkey; serap.karagol@omu.edu.tr

**Keywords:** localization, mobile anchor node assisted localization, obstacle-handling, path planning, static path planning

## Abstract

In many Wireless Sensor Network (WSN) applications, the location of the nodes in the network is required. A logical method to find Unknown Nodes (UNNs) in the network is to use one or several mobile anchors (MAs) equipped with GPS units moving between UNNs and periodically broadcast their current location. The main challenge at this stage is to design an optimum path to estimate the locations of UNNs as accurately as possible, reach all nodes in the network, and complete the localization process as quickly as possible. This article proposes a new path planning approach for MA-based localization called Nested Hexagon Curves (NHexCurves). The proposed model’s performance is compared with the performance of five existing static path planning models using Weighted Centroid Localization (WCL) and Accuracy Priority Trilateration (APT) localization techniques in the obstacle-presence scenario. With the obstacle-handling trajectories used for the models, the negative impact of the obstacle on the localization is reduced. The proposed model provides full coverage and high localization accuracy in the obstacle-presence scenario. The simulation results show the advantages of the proposed path planning model with the H-curve model over existing schemes.

## 1. Introduction

Wireless Sensor Networks (WSNs) are network structures consisting of many small, low-cost sensor nodes that enable information obtained from the Region of Interest (ROI) to be transmitted wirelessly and adapted to many environments. Examples of WSNs application areas include military, health, home, environmental, and industrial applications [1].

Many factors affect the design and performance of WSNs. One of the most important of these factors is localization. Localization can be defined as the process of locating sensor nodes whose locations are unknown in a particular coordinate system. Most localization methods require the location information of some nodes in the network in order to locate the other nodes. The nodes that are known are generally called anchors, beacons, or references in the literature. Ordinary sensors that are the target of localization through the localization process are called Unknown Nodes (UNNs) [2]. For the data collected from the ROI to be meaningful, the sensors’ locations must be known. Accurate sensor position information will also be beneficial for geographic routing algorithms. Device costs should be low as many sensors are used in most WSN applications. In addition, in some implementations, the network will need to be organized without general human management. The sensors may need to work for years or even decades without batteries requiring replacement. Hence, adding a Global Positioning System (GPS) to each device on the network is not energy and cost-efficient in most applications. It is also limited to GPS outdoor applications. Local Positioning Systems (LPSs), on the other hand, use high-capacity base stations deployed in each coverage area, which is a cost burden for most low-configuration WSNs [3].

Depending on the distance measurement method used, localization schemes can be divided into range-based localization schemes and range-free localization schemes. In range-based localization schemes, the distance or angle between the UNN and the Anchor Node (AN) is measured using particular equipment types. The location of the UNN is calculated using these measurement values [4]. Typical range-based schemes include Time of Arrival (ToA), Time Difference of Arrival (TDoA), Angle of Arrival (AoA), and Received Signal Strength Indicator (RSSI) [5]. In range-free localization schemes, it is not necessary to measure physical properties such as distance and angle. These schemes deal with connection information between UNNs and ANs. For example, the number of hops between a UNN and AN can be counted and converted into physical distances. Some examples of range-free schemes are Approximate Point in Triangle (APIT), Centroid, Amorphous, and DV-HOP algorithms. In general, range-based schemes give more accurate results than range-free schemes. However, the structure of range-free schemes is more straightforward; their cost is cheaper and they do not require extra hardware [6].

**Figure 1:**
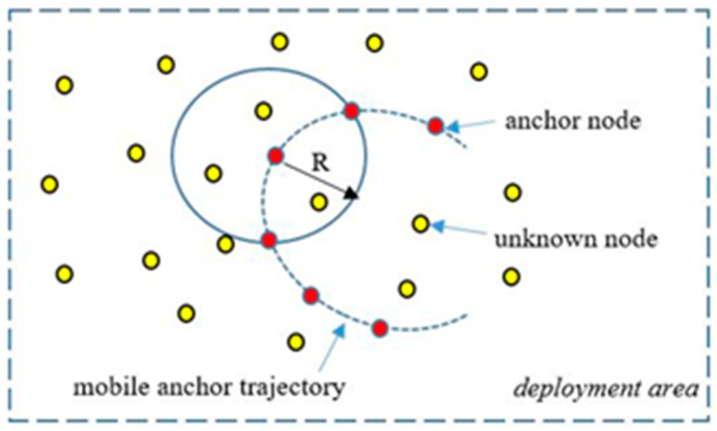
Mobile anchor node assisted localization [7].

Due to the disadvantages mentioned above of GPS for localization in WSNs, approaches generally using as few GPS devices as possible have been studied in the literature. Therefore, it would be more logical to periodically broadcast location information with one or several mobile anchors (MA) equipped with GPS units and position UNNs as shown in Figure 1. On the other hand, unknown static nodes do not move once they are deployed for detection tasks [7].

Since MA’s Mobile Anchor Node Assisted Localization (MANAL) architecture does not have energy constraints like UNNs, its movement trajectory can be carefully designed to improve its localization accuracy. In a network localized with static anchors, a static anchor is not reused after localization. As a result, there will be an extra load for each static anchor not used in the WSN. Compared to a sensor node, a robot is quite large, making it easier to mount the robot’s GPS unit. Hence, MANAL scenarios are more efficient than static scenarios [8].

One of the main challenges in applications using a MA and unknown static nodes is how to design the path the MA will follow. Generally, such a design is aimed at: (i) finding UNNs in the ROI with as few errors as possible when the MA uses the path designed; (ii) finding all nodes in the ROI if possible; and (iii) spending as little energy as possible. In the literature, many studies have been conducted in different scenarios to achieve these and similar objectives. These studies generally addressed essential evaluation criteria such as localization accuracy and did not address realistic channel parameters. In most of these studies, obstacle-free scenarios in the ROI were considered, and obstacle-presence scenarios were not considered. In this paper, a mobile path planning model based on nested regular hexagons (NHex) is introduced for MANAL in WSNs. The proposed model has been designed to consider the criteria for increasing the accuracy and localization ratio of localized nodes for an obstacle-presence scenario. Additionally, realistic wireless channel features are used to evaluate the performance of different mobility models.

In summary, we make the following contributions to a path planning model for MANAL in WSNs:

1. In this study, a new model called NHexCurves is proposed. The proposed model’s different localization performances are compared with the other five models in the regular obstacle environment used in the Z-curve [9] study. This comparison was made on a reliable wireless channel to obtain realistic and fair results.
2. To the best of our knowledge, no study in the literature addresses HILBERT, LMAT, H-curve, and M-curves models with different evaluation criteria in obstacle-presence environments and compares them in these scenarios. In other words, none of the models discussed in this study, except Z-curve, have been performance evaluated in environments with regular obstacles. However, in the Z-curve study, the authors only showed the performance results of their own path planning schemes in the obstacle-presence scenario and did not compare them with the performance of other schemes.
3. Using two localization algorithms, one is range-based, and one is range-free, the variation of the performance of different path models with varying algorithms of localization is examined.
4. In this study, the same localization method was used in the same localization scenario for all path planning models whose localization performance was examined. For example, this is not the case in [10].

The remainder of this paper is organized as follows: in Section 2, mobility and path planning models are classified from general to specific, and then related studies are introduced. Additionally, some localization algorithms used to evaluate path planning models are examined in this section. In Section 3, the network model and its adjustments are given, and the assumptions used in this study are noted. In Section 4, the path planning model proposed in this study and its ideas are introduced. Performance evaluations of the proposed model and some path planning models in the literature in a realistic channel for the obstacle-presence scenario are given in Section 5. Section 6 summarizes results and future work.

## 2. Related Work

**Figure 2:**
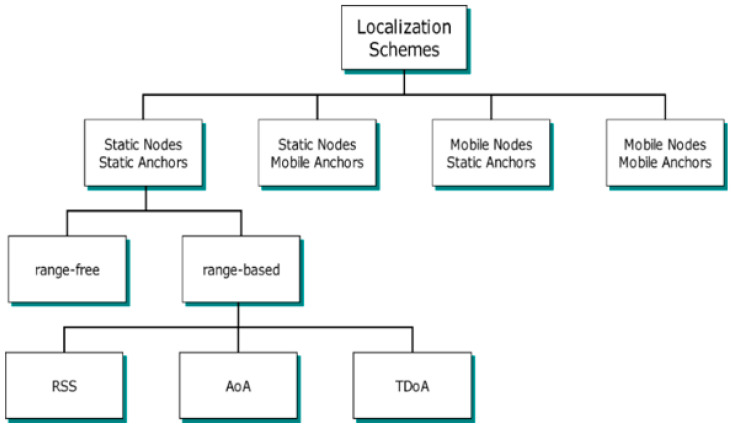
Classification of localization schemes in WSNs [17].

It has become necessary to reclassify the localization schemes, divided into two classes as range-based and range-free according to the distance measurement method, considering the mobility of known or unknown nodes. Therefore, if this classification is expanded to consider mobility, four main classes are obtained, as shown in Figure 2 [11].

In the static anchor static node class, both ANs and UNNs are static. This class is the starting point for the other classes shown in Figure 2. Examples of this class can be found in [12,13]. In the static anchor and mobile nodes class, the estimated location of each UNN will fluctuate around its actual location. As the node will move over time, the old location information will become incorrect. However, the position estimate is revised when updated kernel information is received [14]. An application using UNNs placed in currents along a river and fixed ANs placed on the riverbank to determine these nodes’ locations is an example of this type [15]. In the mobile anchor and static node class, which is also the subject of research in this study, the UNNs are static. MAs roam the ROI and periodically share their location information to UNNs to estimate their location. This kind of localization scheme is also divided into two categories: random mobility and planned mobility. This class will be discussed in detail in Section 2.1. In the mobile anchor and mobile node class, both UNNs and ANs are mobile. An example can be found in [16].

### 2.1. Mobile Anchor—Static Node

In this approach, a node with locational information called MA broadcasts this information periodically, allowing the UNNs locations to be found according to some localization techniques (e.g., RSSI, TOA, or TDOA). The total cost in the network will be reduced by using a single anchor node [18]. The classification of the paths in the approaches finding UNNs with the MA movement and detailed information about these paths are given in the following subsection.

#### Mobile Anchor Paths

**Figure 3:**
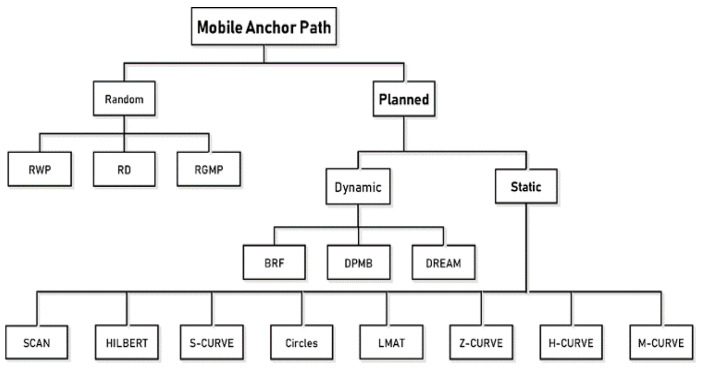
Different path models for MANAL in WSNs.

The studies conducted to design an optimum path model in the literature are categorized in [11,19]. Path models are divided into two main classes as random and planned models, as seen in Figure 3. Random models are often used in applications where high localization errors or high coverage performances are not required. For example, the RWP model is random path mobility in which the direction and speed of the MA are changed randomly, first proposed for routing in ad hoc networks and then modified for use in localization models [20]. Random Direction (RD) and Reference Point Group Mobility (RPGM) are other examples of random path planning models [21].

Planned models can be dynamic or static. In dynamic path planning models, the path is constantly updated with the information obtained on the path according to the need in the application. Therefore, there is no predetermined way. Dynamic Path of Mobile Beacon (DPMB) [22], Breadth-First (BRF) [23], and Deterministic Dynamic Beacon Mobility Scheduling (DREAMS) [24] are some examples of dynamic path planning models in WSNs. These models have a large number of applications in different fields. Some of these are applications such as mobile robots [25,26], autonomous underwater vehicles [27], and autonomous driving [28].

Studies on designing the path followed by MAs include SCAN, DOUBLE-SCAN, and HILBERT [19], localization algorithm with a mobile anchor node based on trilateration (LMAT) [29], Z-curve [9], H-curve [11], and M-curves [10]. These models can be evaluated in the “Static Mobility” subclass of “Planned Mobility”. The model developed in this paper is also a static path planning model because the path, starting point, and endpoint to be followed by the MA are determined before the localization process. These were designed before the MA was released to the ROI. There is no external intervention with respect to the trajectory it follows while the MA is in movement. Intervention while the MAs are moving belongs to the “Dynamic Mobility” class. As the primary goal of static path models is to localize all UNNs, generally speaking, these models have high localization rates and offer low error values compared to other mobility models. Most static path planning models in the literature are based on trilateration and triangulation techniques. Therefore, the designed path should be planned so that the MA provides at least three different location information to the UNNs. However, when a UNN receives a single location information message from one direction or receives multiple location information messages from a different direction on a line, the collinearity problem arises. This is one of the main problems in static path planning models.

**Figure 4:**
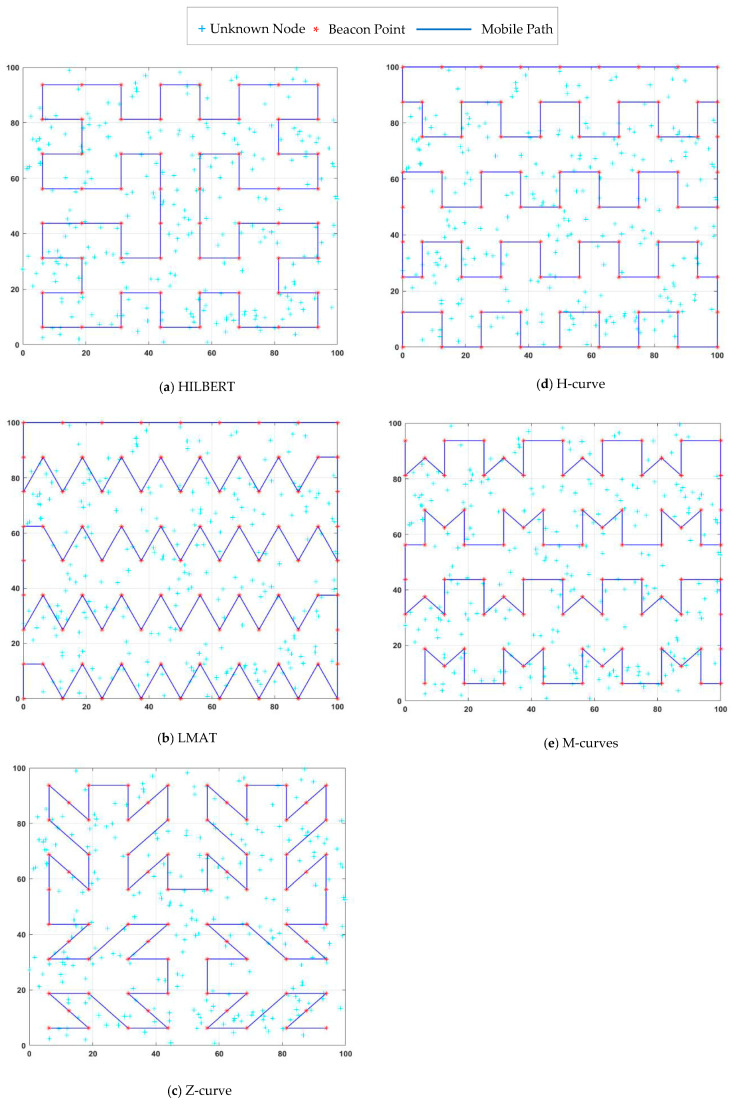
Different mobile path planning models in obstacle-free field.

In [19], SCAN, DOUBLE-SCAN, and HILBERT path planning models were proposed. These models are known as the first mobile-assisted static path planning models in the literature. In the HILBERT path planning model, the idea of making more turns in the trajectory is proposed to solve the collinearity problem of SCAN and the path length problem of DOUBLE-SCAN. For this purpose, the ROI is divided into four squares of equal size, and the points between the squares are joined. UNNs can estimate their location more accurately than the SCAN model using different non-collinear location information from the MA. However, one of the most critical problems of the HILBERT model is the problem of coverage. MA cannot provide sufficient information to UNNs located at ROI boundaries to estimate their location using the HILBERT path planning model. This will increase localization error and reduce the number of localized nodes. The HILBERT path planning model is shown in Figure 4a.

Han et al. [29] presented a path planning scheme called LMAT based on trilateration for MAs. In this scheme, the distance between the two locations in which the MA broadcasts location information is defined as the resolution. MA continues its trajectory by forming symmetrical equilateral triangles and broadcasts location information to UNNs at each vertex of these equilateral triangles. This process ensures that the collinearity problem is solved successfully. High localization accuracy and high localization ratio are achieved with LMAT. However, the length of the path traveled by the MA is long. The LMAT path planning model is shown in Figure 4b.

Rezazadeh et al. [9] proposed a MA path planning scheme called Z-curve. The MA’s path is “Z”-shape. In this study, the authors divided the ROI into squares for three levels and connected the “Z” shapes at each level. As with most mobile path planning models in WSNs, providing three non-collinear points to UNNs in the Z-curve model is one of the main objectives. Additionally, the Z-curve performance is evaluated in the presence of obstacles, and the Z-curve obstacle-handling trajectory is proposed to reduce the obstacle problem in localization. The Z-curve model is shown in Figure 4c.

Alomari et al. [11] presented a MA path planning scheme called H-curve. This scheme is called H-curve because of the multi-curved “H”-shaped paths in the design. The path was designed to solve the collinearity problem, shorten the distance traveled by MA, and cover each UNN with at least three different MA. This study’s critical point is to create a distance difference *d_m_*/2 between two rows, *d_m_* MA’s step interval. Thus, the two rows do not overlap, the number of points decreases, and ultimately a triangle-like communication form is created. Weighted Centroid Localization (WCL) and Weight-Compensated Weighted Centroid Localization (WCWCL) methods were used as localization estimation methods. The H-curve path planning model is shown in Figure 4d.

Kannadasan et al. [10] devised a different path planning scheme that follows MA’s trajectory “M” shape. Therefore, they called the proposed scheme “M-curves”. In this study, two successive rows are patterned with the letters M and W. There is a *d_m_*-long space between the letters M or W in each row. A *d_m_*/2 distance difference is created between two rows. Thus, a triangle-like communication form was obtained, similar to the H-curve, and a solution was found to the collinearity problem. The authors used the “Centroid Method” as the localization estimation method and aimed to increase the performance of the proposed method by adding the “Dolphin Swarm Algorithm (DSA)”. The M-curves path planning model is shown in Figure 4e.

Some static path planning schemes proposed in the literature have been designed and tested in obstacle-presence scenarios. For example, in [30], Magadevi et al. presented obstacle-avoiding localization algorithms based on the path planning scheme called V-curve. The main curve of the V-curve path planning mechanism is the combination of SCAN and Z-curve. In this study, the presence of static and dynamic obstacles in the sensing field was examined. If a repulsive force is applied to the MA by the obstacle, it will use the backtracking technique until it reaches the previous node *Sj* successfully visited. After a delay, it moves back into the V-Curve path. If the problem recurs, it will follow along an edge of the *Sj* node. In [31], Han et al. proposed a new path planning algorithm called SLMAT, combining LMAT and SCAN algorithms to enable a trade-off between location accuracy and energy consumption. In this study, the authors used the MA for disaster management. Therefore, the necessity of the MA to avoid obstacles on the path was established. The MA moves along a predefined trajectory and broadcasts beacon messages at beacon points. When an obstacle is detected, the MA will move around the obstacle and broadcast location messages on roundabout flags.

**Figure 5:**
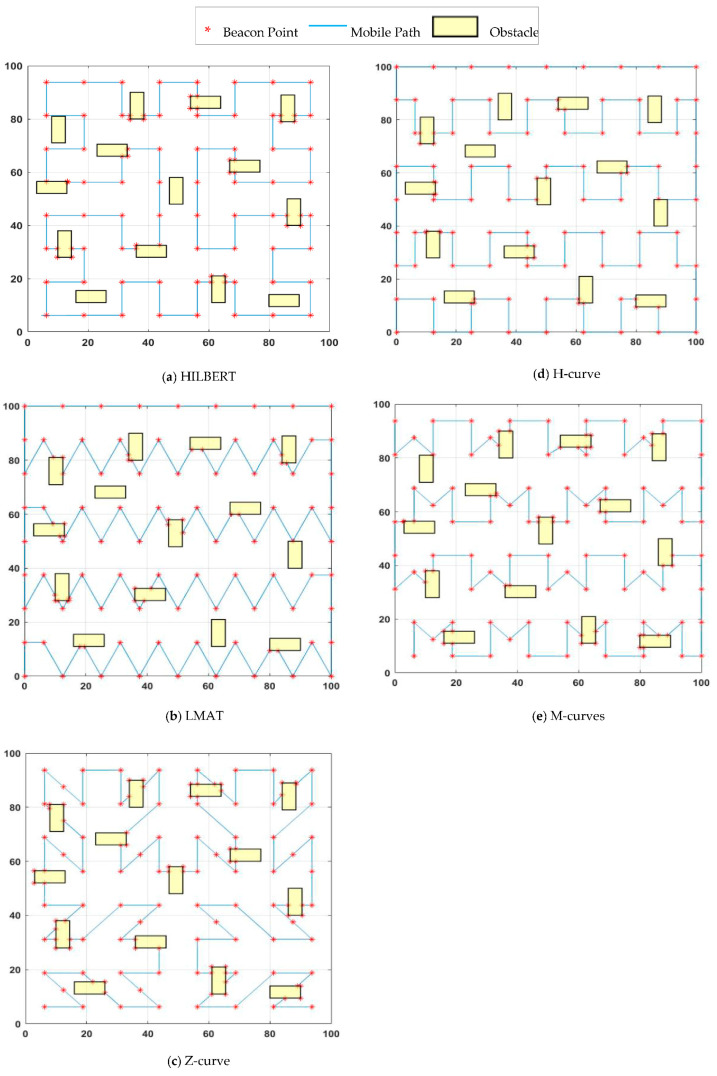
The obstacle-handling trajectories of the path planning models.

As mentioned, the collinearity problem, which is one of the most fundamental problems in designing static path planning models, stands out as the first condition to be addressed while developing a model. SCAN, DOUBLE- SCAN models were limited by this problem. The authors overcame this with the HILBERT model. However, this model also faces a coverage problem as it cannot reach the nodes at the corners of the ROI. Therefore, coverage is another of the conditions that should be considered in the design. Reliable channels should be used to achieve realistic results. If a static path planning model achieves successful results with different localization methods, it is adaptable to different environments. However, this was not demonstrated in the M-curves study. Although path length does not directly affect the localization error rate or the number of localized nodes, it helps determine the time required to complete the localization process. It can also affect other critical metrics such as energy consumption. Therefore, the path length of the designed path planning model should be as short as possible. The LMAT model is a path planning model with good localization performance in general. However, the path length is quite long. Additionally, note that the studies’ performance evaluations in Figure 4 in the presence of obstacles have not been studied, except for Z-curve. This study’s main contribution is to measure the performances of the proposed scheme and the path planning schemes in Figure 4 in the presence of obstacles and compare them using different evaluation criteria. All path planning models have been tested in the same environment, and this environment is the obstacle-presence environment where a third-order Z-curve is applied [9]. The obstacle-handling trajectories of the path planning models in this environment are given in Figure 5. Detailed information on the design stages of the path planning model proposed in this study and the comparison of the model’s error performance in the obstacle-presence environment with the known and strong static path planning models in the literature will be given in the following sections. In addition, the localization performance of the model proposed in this study at low resolutions is superior to other models. The model, which gives very good results in high resolution, can give very bad results in low resolution. The opposite can also be seen. However, there are no major differences between high-resolution localization performance and low-resolution localization performance in the proposed model.

### 2.2. Localization Schemes

Another important research topic in MANAL algorithms is to design a localization scheme where UNNs locations are calculated using MAs. Localization schemes are an essential criterion in evaluating the performance of mobile path models. If a mobile path planning model works well in scenarios using different localization schemes, it can be said that this model is compatible with working in different locations. In the literature, in general, trilateration and triangulation-based localization schemes were used to evaluate mobile path models. RSSI and TOA approaches are generally preferred for these schemes.

In this study, since Weighted Centroid Localization (WCL) and Accuracy Priority Trilateration (APT) schemes are used, these schemes are examined separately in the following subtitles.

#### 2.2.1. Weighted Centroid Localization (WCL)

WCL is a localization method where the locations of UNNs are calculated using the average of the coordinates of the location information they receive from the beacons. Communication consumption and computation cost are low. In the WCL method proposed in the study [32], a *w_ij_* weight function was defined based on the RSSI value that varies according to the distance between the UNN and the AN to examine each *B_j_*(*x*,*y*) coordinate information’s effect received. *w_ij_* can be calculated as:

(1) $$w_{ij}=\frac{1}{{\left(d_{ij}\right)}^{g}}$$

where *g* is a default degree for different situations; thus, the node location can be formulated as follows (indicates the number of beacons received from the anchor):

(2) $$P_{i}=\frac{\sum_{j=1}^{n}w_{ij}B_{j}\left(x,y\right)}{\sum_{j=1}^{n}w_{ij}}$$

After replacing *w_ij_* with *RSSI_ij_*, the final equation is obtained as:

(3) $$P_{i}=\frac{\sum_{j=1}^{n}{RSSI}_{ij}B_{j}}{\sum_{j=1}^{n}{RSSI}_{ij}}$$

#### 2.2.2. Accuracy Prıority Trilateration (APT)

**Figure 6:**
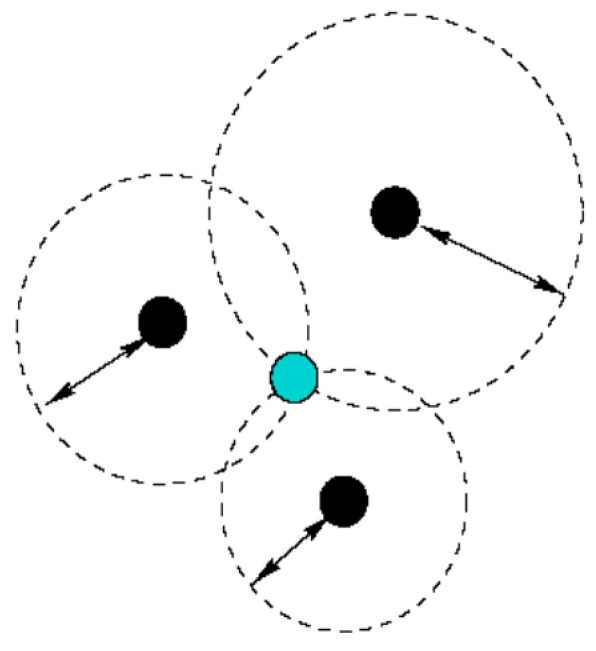
Trilateration technique.

The trilateration method is a geometric technique that uses distances between three ANs and one node of the unknown location to determine a UNN’s location. A UNN can be localized when at least three reference points associated with it in two-dimensional space are known. The location of the UNN can be estimated by calculating the intersections of three circles. This situation is shown in Figure 6. In this way, three dark-colored nodes represent the ANs whose locations are known, and the light-colored node represents the target node whose location information is trying to be obtained by using the positions of the ANs [33].

With the APT technique, the UNN location is obtained by using the three closest location message information from the MA to the UNN. In this technique, it is possible to estimate the UNN with high accuracy since three AN values closest to the UNN are considered [9].

## 3. Network Settings and Assumptions

The network model used in the path planning model proposed in this study is assumed to have the following features:

1. The UNN and the MA are two types of sensor nodes in the network. *N* number of UNNs are deployed randomly to the network with uniform distribution.
2. ROI is represented by a two-dimensional area, and a WSN with an S = 100 × 100 m^2^ area size has been established.
3. After the UNNs are distributed to the environment, their location information is not known at the first stage. It is assumed that all of these nodes are static, and their positions are unchanged.
4. The communication range (CR) of each sensor node in the network is fixed, and its value is CR m.
5. An MA is location-aware within the network and is ready to traverse the entire network in straight lines depending on each path pattern.
6. The MA stops at certain intervals while moving. It is preset as dx or $\mathrm{dx}/\sqrt{2}$ m between two successive points. The difference between intervals will be explained in Section 4.1.
7. The MA sends the location signals from its stopping points to UNNs within the CR. Both the MA and the UNN can communicate with each other only if both are in each other’s CRs.
8. The distances from UNNs to MAs are estimated using the RSSI technique. When the UNN receives at least three non-collinear localization messages, it determines its location according to the APT localization method. In the WCL method, all ANs in the CR are taken into account.
9. The MA has enough energy to move and broadcast location information during the localization process. MA consumes more power than any UNN.
10. The communication model is a channel by log-normal shadowing fading. Considering the radio signal propagation loss, we assume that unknown nodes can only receive the beacon packet in a circular region within a communication radius.
11. There is no collision between the MA and the UNNs; that is, MA does not go through any UNN.

## 4. Proposed Work

**Figure 7:**
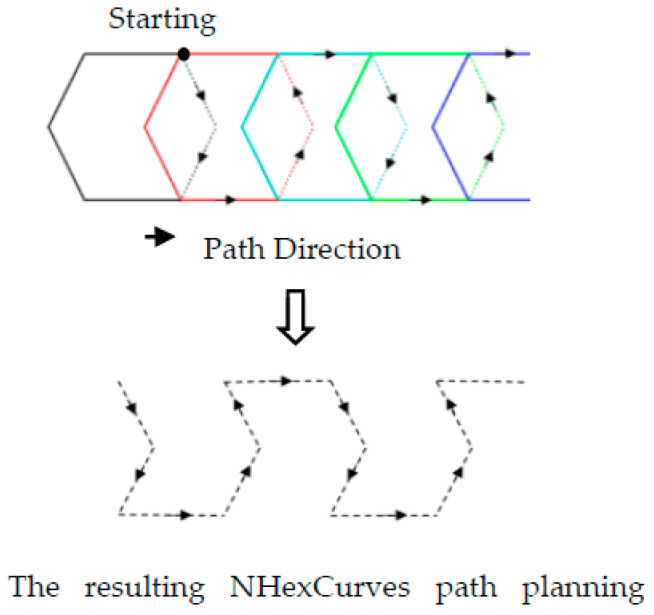
Nested hexagons and obtaining the NHexCurves pattern.

Designing an optimum MA trajectory for the localization problem in mobile WSNs is a significant challenge. For this problem’s solution, the model proposed in this study is called nested hexagon curves (NHexCurves) because the pattern is obtained by intersecting regular hexagons, as in Figure 7. NHexCurves is designed such that any UNN can receive non-collinear information messages from three different beacons. As such, the MAs between the two NHexCurves patterns form an isosceles triangle. The beacon points in an NHexCurves model also include two differently sized isosceles triangles. Therefore, high coverage and strong localization accuracy can be achieved.

### 4.1. MA’s Mobility Phase 

**Figure 8:**
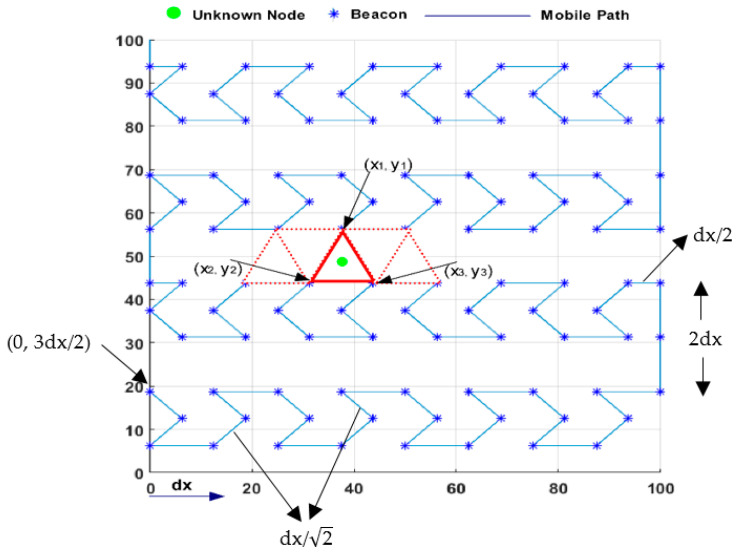
The proposed anchor path trajectory—NHexCurves.

In this study’s path planning model, the MA starting from (0, 3dx/2) point follows the dx length horizontal segments of the pattern and the $\mathrm{dx}/\sqrt{2}$ length cross line segments of the pattern with straight lines, as shown in Figure 8. The MA that completes a row movement moves up to 2dx higher on the y-axis and starts moving in the opposite direction of its previous movement. The MA begins drawing the NHexCurves pattern designed in this row after a straight line of dx/2. This process prevents two consecutive lines from intersecting, ensuring an isosceles triangle between the two rows and thus eliminating the problem of collinearity. The MA, which completes its pattern at the end of the second row, moves up to dx this time and continues the same process until it covers the entire ROI. If we look closely, the second row is completed by continuing the logic of creating the same triangle form of communication.

### 4.2. Mobile Anchor—Unknown Node Communication and Localization Estimation Phase

For this phase, let us first consider the APT scenario. In the case where three different beacon points, such as (x_1_, y_1_), (x_2_, y_2_), and (x_3_, y_3_), are obtained as in Figure 8, one of the UNNs in the CRs of these beacons can estimate their position with the information from the beacons. What is important here is that these three beacons are not collinear. If less than three non-collinear localization information is received, the UNN will wait until the MA provides the expected number of non-collinear localization information. UNNs are expected to receive localization information from three non-collinear points using the triangles of different sizes created in the mobile path model proposed in this study. When a UNN receives three different non-collinear localization messages, it is ready to start its own location estimation process using APT.

Secondly, when we consider the WCL scenario, the beacons covering each UNN are determined, and the RSSI values varying from each of these beacons to the UNN are calculated. The weight function of each beacon is determined according to the RSSI values obtained. A UNN estimates its position with these values using the WCL method.

#### Obstacle-Presence Scenario

There may be obstacles in the network area in a realistic environment, blocking the MA’s path. In the obstacle-resistant trajectory developed in [34] and used in [9], MA detours the obstacle and issues a detour flag at the obstacle’s corner points. A UNN uses these flag signals to determine its location. When the MA moves away from the obstacle, it returns to the original trajectory and starts broadcasting standard beacon messages. Since the path planning models discussed in this study are deterministic, the movement pattern and beacon positions for message broadcast are already known. Therefore, the MA can cross the edge of the obstacle and continue the path planning pattern where it left off.

**Figure 9:**
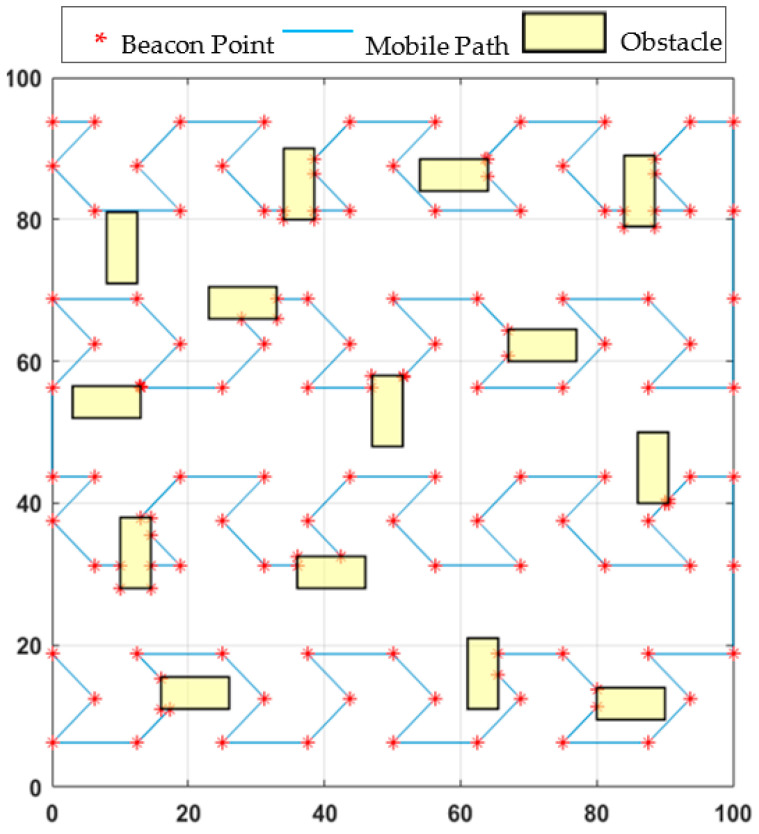
NHexCurves in obstacle-handling trajectory.

In this study, “obstacle-handling trajectory” detailed in [9], which is briefly mentioned above, is used for all path planning models in case of obstacle-presence. The obstacle-handling trajectory determined by applying this logic to the proposed NHexCurves path planning model can be seen in Figure 9.

## 5. Performance Evaluations

**Figure 10:**
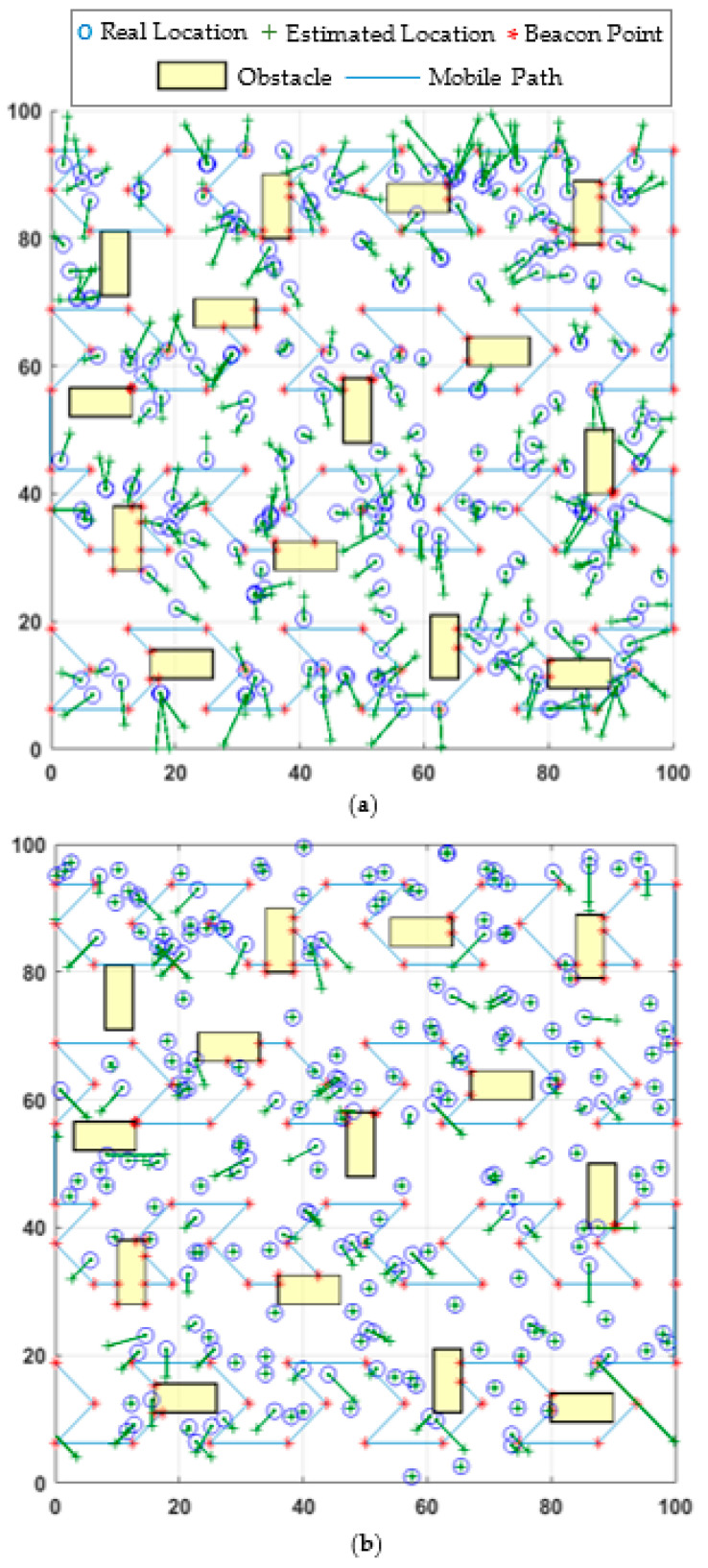
The proposed mobile path’s obstacle-handling trajectory and estimated locations of the nodes distribution in (**a**) WCL, and (**b**) APT.

In this study, five static path planning models are used to compare the proposed model’s performance in the obstacle-presence scenario. These models are HILBERT, LMAT, Z-curve, H-curve, and M-curves. APT and WCL localization algorithms are used to evaluate the various performances of mobility models. Figure 10 shows the obstacle-handling trajectory of the proposed model and the estimated positions of the UNNs in the obstacle-presence scenario using both localization methods, respectively.

### 5.1. Simulation Setup and Wireless Channel

A realistic wireless model is required to make a reliable assessment. The Z-curve simulations [9] consider the modulation, channel model, and coding scheme to obtain the relationship between transmission power and packet receiving rate. Chipcon CC1000 radio module was used in the Z-curve study. Similarly, the H-curve [11] is another study using realistic parameters. For the H-curve wireless model, a wireless node’s characteristics with the Chipcon CC1100 radio module [32] were used. This study also used the wireless channel model and its parameters described in [9]. Explanations and specifications for this channel can be found in the following paragraphs. It should also be noted that all the path planning models in this study are run under the same realistic channel conditions.

The strength of the signal emitted in the physical environment decreases due to wireless propagation. Therefore, path loss and bit error rate should be modeled to analyze the physical layer [35,36]. *P_rr_* packet reception ratio means the possibility of a packet being successfully received. It is expressed by the Bernoulli random variable that takes the value 1 if the packet is received and 0 if not. It is given by:

(4) $$P_{rr}={\left(1-P_{be}\right)}^{8l}{(1-P_{be})}^{8\left(f\text{-}l\right)2.0}$$

where *f* = 20 byte is the frame’s size related to the TinyOS application after being encoded (the frame consists of a preamble, network payload, and CRC). The Manchester encoding method is used; *P_be_* is the bit error probability, which depends on the modulation type. Here, we chose non-coherent FSK modulation, which is used in MICA2 motes and formulated by [35]:

(5) $$P_{be}=\frac{1}{2}e^{\frac{{SNR\times B}_{N}}{2R}}$$

where *B_N_* is the noise bandwidth and *R* is the data rate in bits. MICA2 motes use the Chipcon CC1000 radio [37] where *R =* 19.2 kbps and *B_N_ =* 30 kHz. The signal to noise ratio (SNR) at the receiver is calculated by:

(6) $${SNR}^{dB}=P_{rec}{}^{dB}-P_{n}{}^{dB}$$

*P_rec_* defines the reception power, and *P_n_* expresses the noise floor which is both environmentally- and radio-dependent [38]. It is given by:

(7) $$P_{n}=\left(F+1\right){kT}_{0}B_{N}$$

where *F* = 13 dB is the noise figure, and k is the Boltzmann’s constant. $T_{0}$ = 27 °C is the ambient temperature, and *B_N_* is the equivalent bandwidth. In this study, the average noise floor considered is approximately −105 dBm, which has a difference of 10 dBm with the analytically calculated value in [36]. On the other hand, *P_rec_* is given as:

(8) $$P_{rec}{}^{dB}=P_{trans}{}^{dB}-P_{L}{}^{dB}$$

where $P_{L}{}^{dB}$ and $P_{trans}{}^{dB}$ are power loss and transmitting power, respectively. To model the shadow path loss effect, the most commonly used log-normal model [38] is used:

(9) $$P_{L}{\left(d\right)}^{dB}=P_{L}{{(d}_{0})}^{dB}+10\gamma \log\left(\frac{d}{d_{0}}\right)+X_{\sigma }{}^{dB}$$

where $P_{L}{\left(d\right)}^{dB}$ is the power loss after the signal propagates over distance *d*, $P_{L}{{(d}_{0})}^{dB}$ is the power loss at the reference distance *d*_0_, and γ is the path loss exponent. $X_{\sigma }=N\left(0,\;\sigma^{2}\right)$ is a Gaussian random variable with mean 0 and standard deviation (std) σ (shadowing effect).

In this paper, the performances of different path planning models are evaluated in a MATLAB environment with 50 runs in an obstacle-presence scenario. It is assumed that there are N = 250 static UNNs in the ROI, a single MA moving on a given path, and the ROI is an area of S = 100 × 100 m^2^. The resolution value (R) is the ratio between the CR and the MA step dx. That is, the resolution is expressed as CR/dx. 

Other parameters used in this study are listed in Table 1.

### 5.2. Accuracy

One of the most important criteria for evaluating any proposed model is the accuracy of localization. Therefore, this criterion is considered the primary criterion used to compare different path planning models. Accuracy is measured by the number of localization errors in the nodes. In this study, two methods are used to calculate each model’s localization error: the average localization error and the std of the localization error.

#### 5.2.1. Average Localization Error

The average localization error is used to assess how accurate the estimated position obtained is. The ratio of the sum of the localization errors of all the localized UNNs in the ROI to the number of nodes determines the average error rate. The localization error for node *i* is calculated as follows:

(10) $${\mathrm{error}}_{\left(i\right)}=\sqrt{{{(x}_{i}-u_{i})}^{2}+{{(y}_{i}-v_{i})}^{2}}$$

(x_i_, y_i_) are the actual coordinates of the node discussed and (u_i_, v_i_) are the estimated coordinates of the same node. Therefore, error_avg_ is given by the average localization error equation for the total N sensor nodes.

(11) $${\mathrm{error}}_{\mathrm{avg}}=(\overset{N}{\underset{i=1}{\sum }}{\mathrm{error}}_{\left(i\right)})/N$$

**Figure 11:**
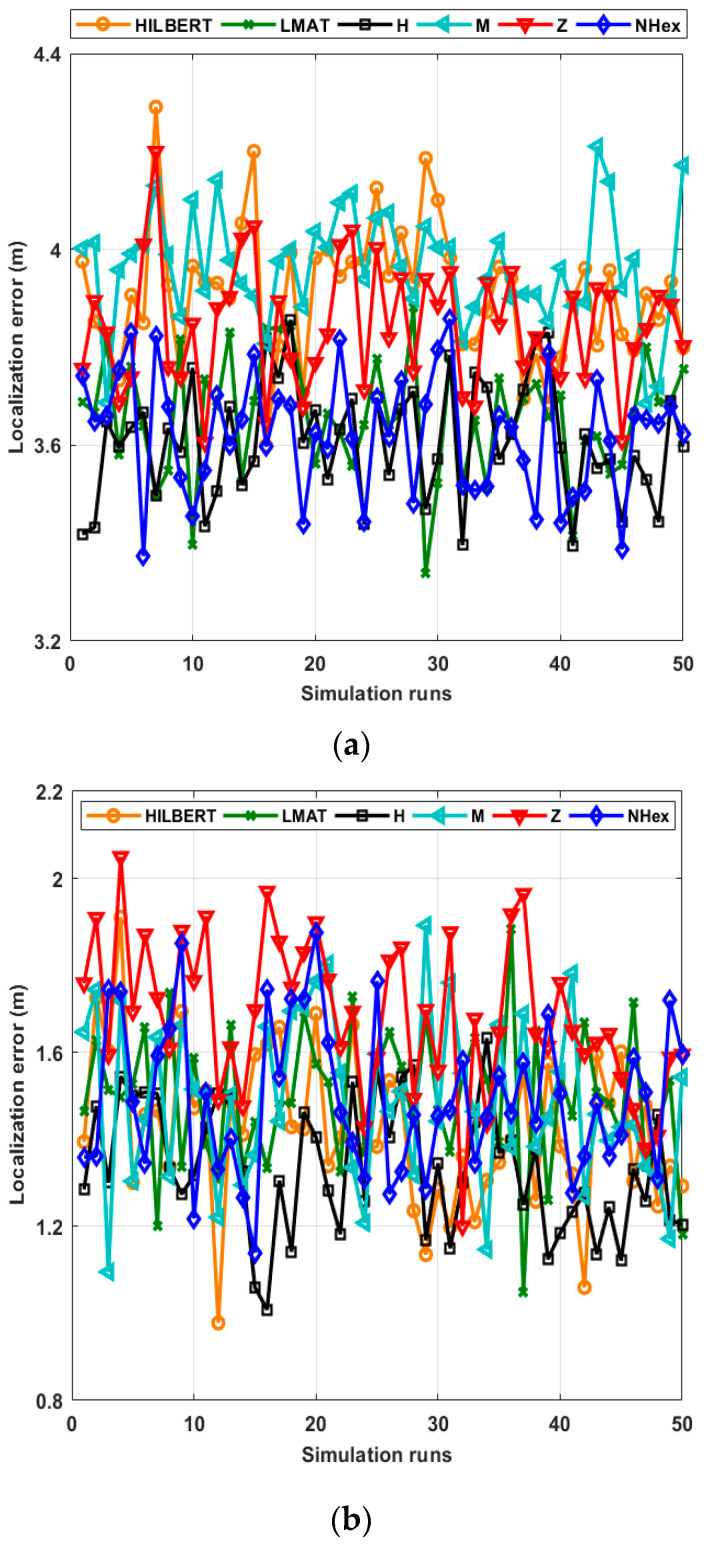
Localization errors of all mobile path planning models in (**a**) WCL, and (**b**) APT.

However, in order to compare the average localization error in each path planning model discussed, performance testing is first performed in an environment with 250 UNNs with a CR of 12.5 m. The resolution value is taken as *R* = 1 and the std of the noise as *σ* = 7. Figure 11 shows the performance of path planning models for 50 simulations in terms of the localization error. As shown in the figures, the models’ error performances obtained are close to each other. For this reason, to establish more accurate error analysis, statistical characterization of localization errors of path planning models has been made. Table 2 and Table 3 show the statistical characterization of different static path planning models for WCL and APT, respectively.

**Figure 12:**
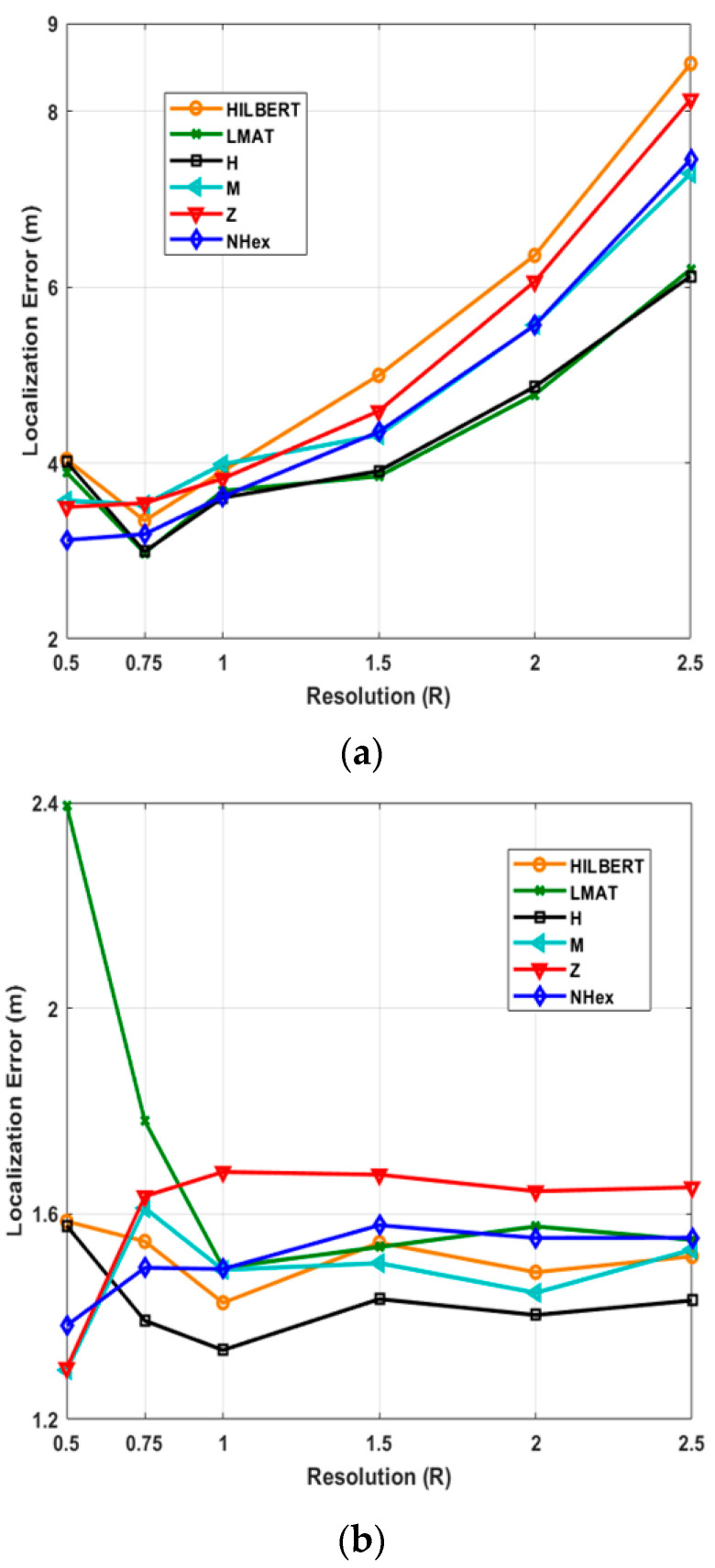
Average localization errors of models by resolution in (**a**) WCL, and (**b**) APT.

As can be seen in Table 2, according to most statistical evaluation criteria, the H-curve path planning model showed the best error performance compared to other models. After this model, the best error results are obtained with the proposed NHexCurves and LMAT models, respectively. While the other models’ error performances are similar, HILBERT model is noticeably worse than the other models. If we pay attention to the path trajectories of the H-curve and LMAT models in Figure 4, it is seen that the MA travels the boundaries of the ROI. Hence, in these models, MAs share the beacon signal at the borders. This is why these models localize UNNs with less error. In the proposed model NHexCurves, beacons are shared on both sides of the ROI. At the same time, since there are too many turns in this model, the number of beacons increases with the shared beacons when they hit the obstacle. Therefore, the localization error of the proposed model is also low. The HILBERT model does not reach the borders of the ROI. Thus, a higher error rate is obtained. In Table 3, the same test is performed using the APT localization method. Generally, it is observed that the performance of path models improved when this method is used and this is the expected result. Since accuracy is the priority in APT, three UNNs closest to the beacon are considered. Therefore, it is possible to achieve higher accuracy in results. In this scenario, although the path planning models’ error performances are similar, the H-curve gives the best result, and the Z curve contains slightly more error than the others. The reason why the Z curve performs the worst in this scenario is that the resolution is *R* = 1, and the localization method is APT. As mentioned, APT uses the three closest beacons for localization. However, there are places with long distances between two beacons in the Z-curve trajectory. Therefore, the resolution should be low for the Z-curve to give good results in APT. This situation can be seen in Figure 12b.

In this study, the average localization error variation with two variables given in Table 1 is also investigated separately: resolution (*R*) and std (*σ*). Firstly, 250 UNNs simulations with different resolutions ranging from 0.5 to 2.5 are performed. Figure 12 shows the average localization error according to the resolution values for path models and two different localization algorithms.

As can be seen from Figure 12a, the proposed NHexCurves model showed good error performance at low-resolution values for the WCL method. The proposed model stands out compared to other models, especially with a value of *R* = 0.5. As is known, WCL takes the weighted average of the beacons in the coverage area to determine the locations of UNNs. If paying attention, there are many turns in the proposed model. Beacons are also published on these turns. This increases the probability of finding more beacons than the uniformly distributed ROI for the WCL method, especially when the resolution decreases. Therefore, the performance of the proposed model can be higher at low resolutions. After *R* = 1, the H-curve and LMAT models showed the best error performance. NHexCurves comes after these models. The performance of the H-curve and LMAT models are similar. In general, error values are obtained slightly higher due to the WCL method. The worst result is again achieved with the HILBERT model. Figure 12b shows the results of the same scenario for the APT method. Here the error values are generally similar, and far fewer errors are obtained than in the WCL. In the proposed model, the distance between the beacons is less than the other models. This is because NHexCurves contains more triangles in different sizes than other models. For this reason, NHexCurves show high performance at low resolutions. Additionally, interesting results have been encountered in this scenario. It has been observed that the LMAT model, which shows stable results after *R* = 1, gives higher localization error at resolution values lower than this resolution value compared to other models. Although different step intervals are used in other models, if we pay attention, the step range of LMAT is fixed and 12.5 m, and LMAT uses equilateral triangles in its path model. Therefore, in APT, the localization performance of LMAT is low for resolutions less than *R* = 1. The Z-curve model, which generally provides higher localization error results than other models, gave the best error result with M-curves at *R* = 0.5. The performance of the proposed NHexCurves model is also very close to these models. However, it can be said that the H-curve gives the best error performance in the overall performance evaluation in this scenario.

**Figure 13:**
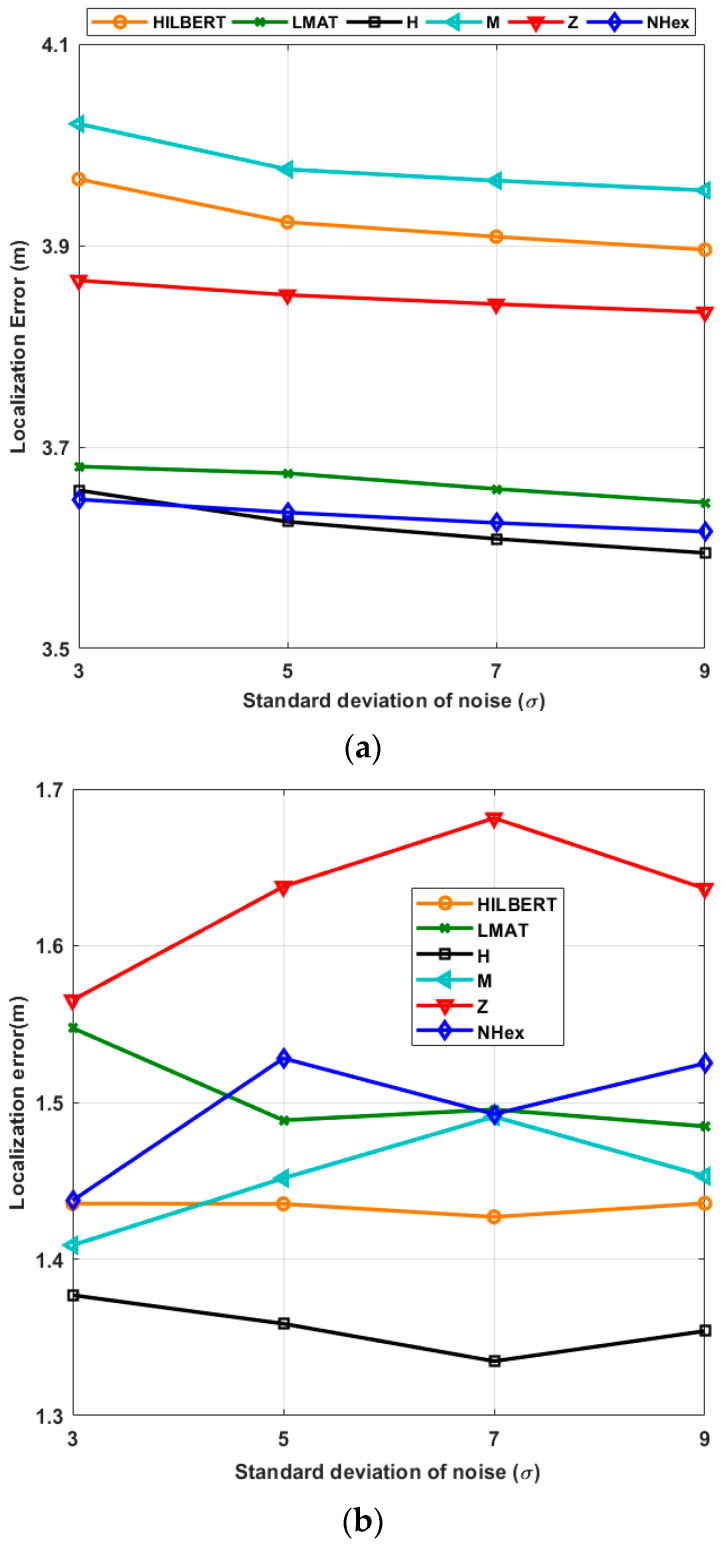
Average localization errors of the models according to the std of the noise (*R* = 1) in (**a**) WCL, and (**b**) APT.

Secondly, in Figure 13, the same parameters are repeated with different std of noise (*σ*) values for the fixed resolution. Figure 13a shows the average localization error for the path planning models with a different *σ* when the WCL algorithm is applied, and Figure 13b illustrates the same as when APT is used. In the first algorithm, the model with the smallest localization error for all σ values is H-curve. Additionally, the error performance of the proposed model obtained is very close to H-curve in this scenario. For these two models, the error values *σ* = 3 and *σ* = 5 are almost equal. Compared to the other models, it can be said that the error performance of the proposed model changes less with changes in *σ*. The reason that the localization error does not change much with the *σ* is characteristic of the WCL method that takes the weighted average of the beacon locations. As can be seen from Figure 13b, H-curve, M-curves, and HILBERT path planning models provided a slight superiority compared to others. If you pay attention, the results obtained for all models are very close to each other, and the APT method again provided more precise accuracy than WCL.

#### 5.2.2. Standard Deviation of the Localization Error

The low std of error values in a data set indicates that most of the data set are close to the mean. The std of the localization error is represented as follows:

(12) $${\mathrm{error}}_{\mathrm{std}}=\sqrt{\frac{1}{N}\overset{N}{\underset{i=1}{\sum }}{\left({\mathrm{error}}_{\left(i\right)}-{\mathrm{error}}_{\left(\mathrm{avg}\right)}\right)}^{2}}$$

where N is the number of localized nodes, error_(i)_ is the localization error for node i, and error_(avg)_ is the average localization error.

**Figure 14:**
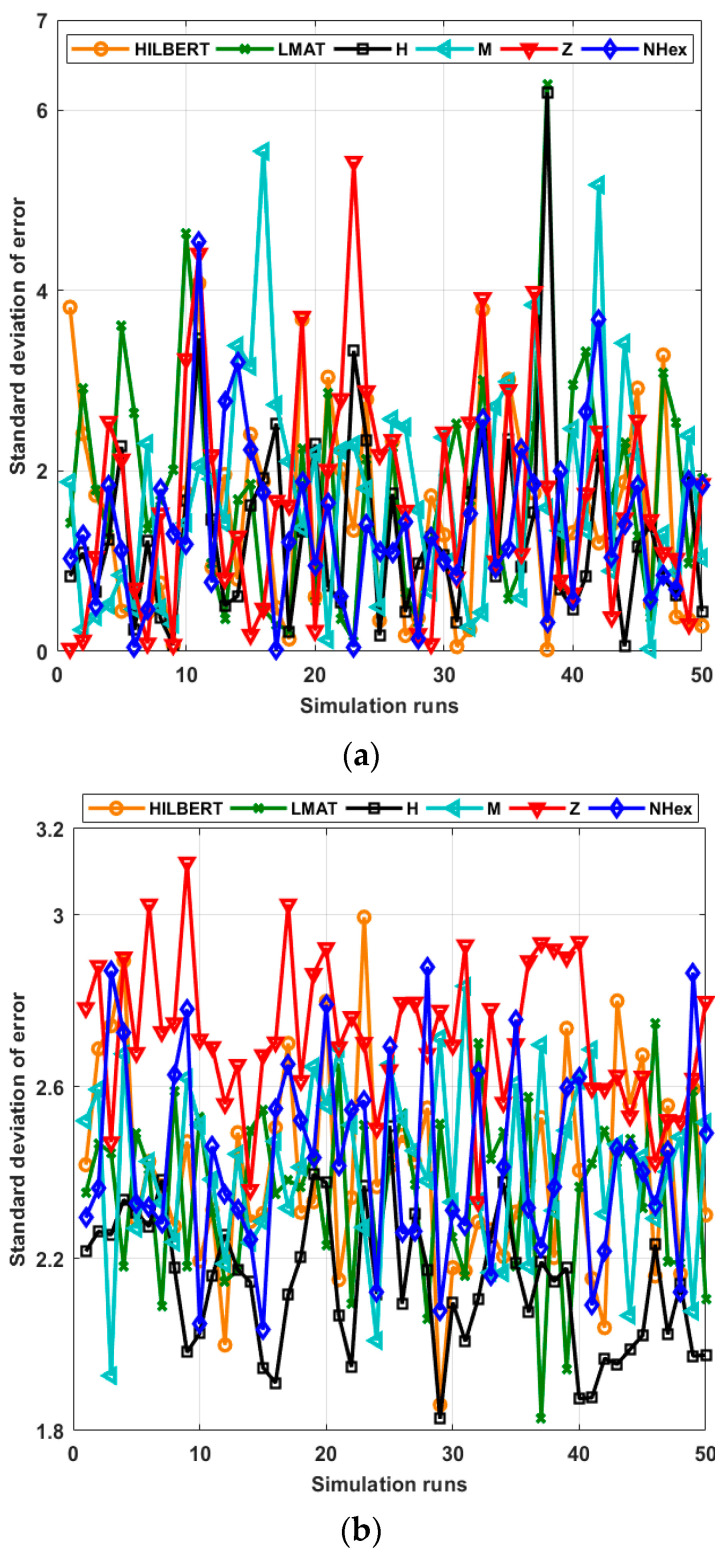
Std of errors of all path planning models in (**a**) WCL, and (**b**) APT.

Figure 14a shows the std of the localization error for each path planning model for WCL and Figure 14b for APT. *R* = 1 and *σ* = 7 for each simulation. Since the std of localization error performance of the errors according to the number of simulation runs was close, as in Figure 11, statistical characterization is again preferred to facilitate performance interpretation of the considered path planning models. Table 4 and Table 5 show the statistical characterization of different static path planning models for WCL and APT, respectively. As can be seen from Table 4, the std of error performance of the H-curve and NHexCurves path planning models for the WCL method is better than the other models, and the performances of these models are similar. According to this performance criterion, the LMAT model’s performance is relatively poor compared to the others. When the same evaluation is performed for the APT localization technique, it can be said that the H-curve model stands out compared to other models. The Z-curve model has been the worst-performing method in this scenario.

**Figure 15:**
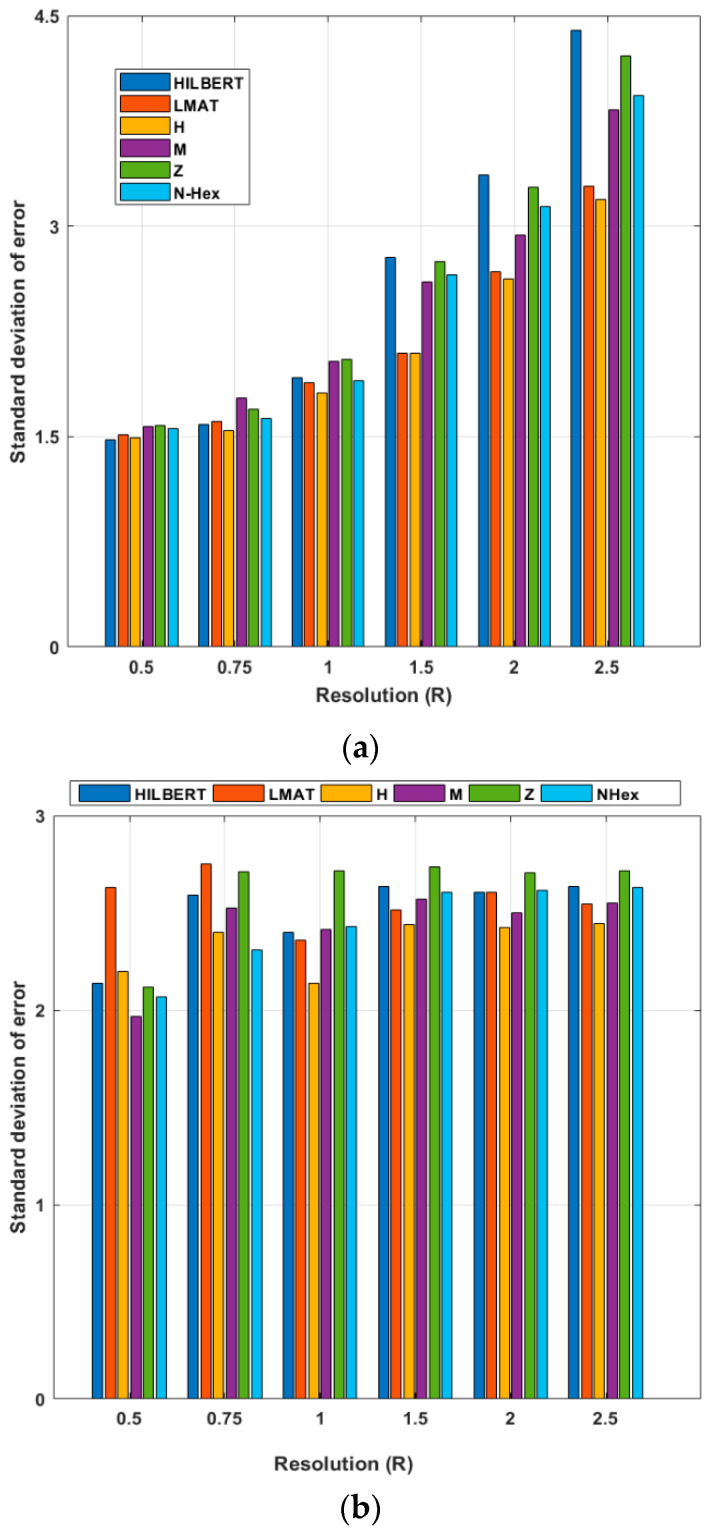
Std of errors of the path planning models according to resolution (σ = 7) (**a**) WCL, and (**b**) APT.

The average of the 50 simulations is then calculated using different *R* values, as shown in Figure 15. In Figure 15, σ = 7 and *R* varies between 0.5 and 2.5. These figures show the std of the localization error for different resolution values. When the performances of the models for the WCL method are evaluated, the error performances of the models at *R* = 1 and smaller resolution values are generally close to their average. However, at larger resolutions, the error performance of the models is slightly different from the average. As the resolution increases, the std of the error value of the models increases according to the previous resolution. The std of error performance of the proposed path planning model is higher at lower resolutions. For the WCL method, the LMAT and H-curve models show the best std of the error performance. According to the same evaluation criteria, HILBERT again shows the worst result. When the same error performance evaluation of the models for the APT localization method is made, it can be said that the proposed NHexCurves model shows the best performance at resolution values less than *R* = 1. It should be noted here that the LMAT model, which is one of the models that gives the results of the std of the best error for the WCL method, gives poor performance, especially at low resolutions (*R* < 1) in the APT method. As shown in Figure 12b, the LMAT show high error results for *R* < 1. After *R* = 1, it can be said that the H-curve is the best performing model and the Z-curve model is the worst performing model, although there are no significant differences between the performance of the models in general.

### 5.3. Localization Ratio

**Figure 16:**
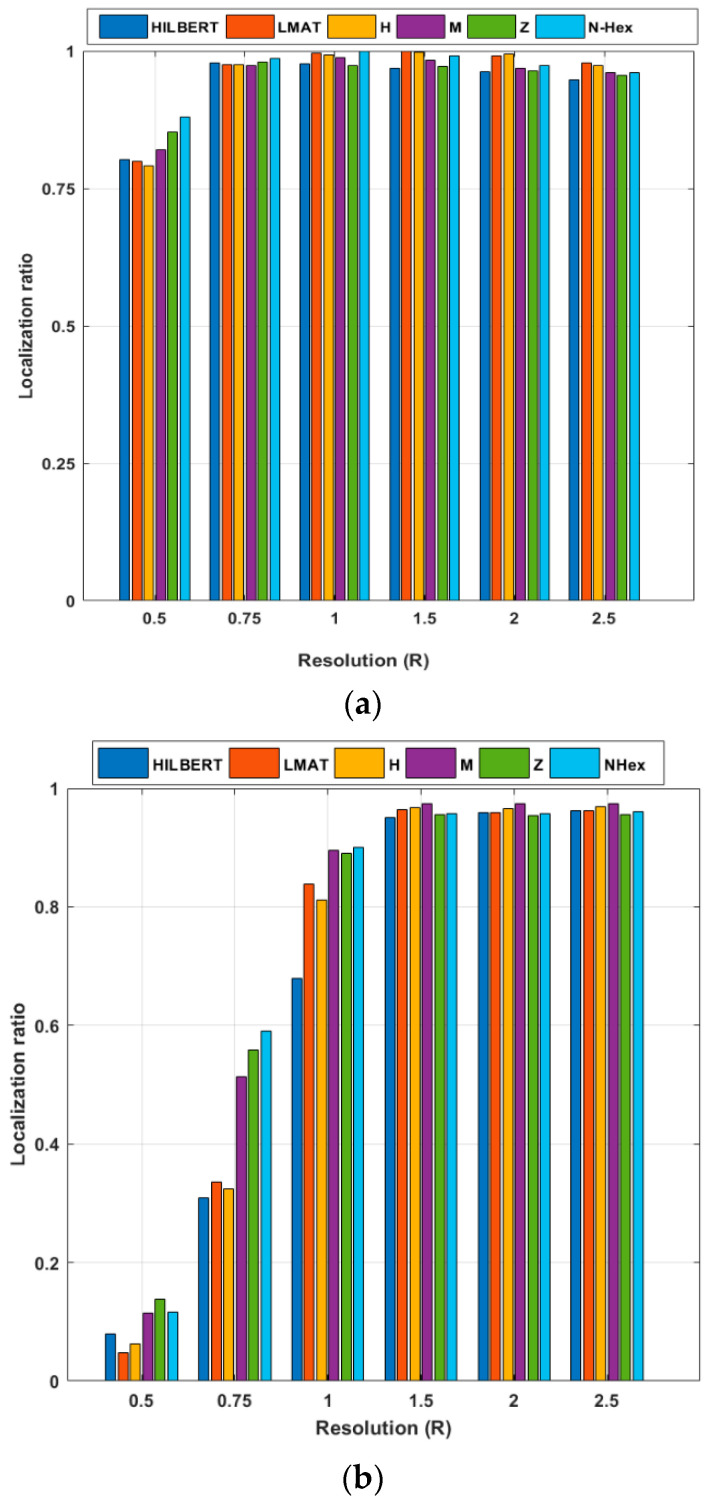
Localization ratio of the path planning models by resolution (σ = 7) (**a**) WCL, and (**b**) APT.

In the scenario created in Figure 16, the localization ratios of the models in different resolutions for 250 UNNs are examined for the constant σ = 7 value. If the localization ratio performances of the models for the WCL method are evaluated, the model that gives the highest localization ratio performance at low resolutions (*R* < 1) is the proposed NHexCurves model. For other resolution values, it can be said that all models show high performance in general. However, the localization ratio performance results obtained from the simulations made with the APT method are different. It can be said that the proposed NHexCurves model for *R* ≤ 1 performs slightly better than other models. HILBERT model showed the worst localization ratio performance for all resolution values except *R* = 0.5. This is because HILBERT cannot reach the nodes in the corners of the ROI. The APT method observed that for *R* = 0.5 and *R* = 0.75, the LMAT method localized very few nodes and showed worse results than other methods. For *R* > 1, although the localization ratio performance of all models is high and close to each other, it can be said that the M-curves model stands out, albeit a little, here.

### 5.4. Scalability

**Figure 17:**
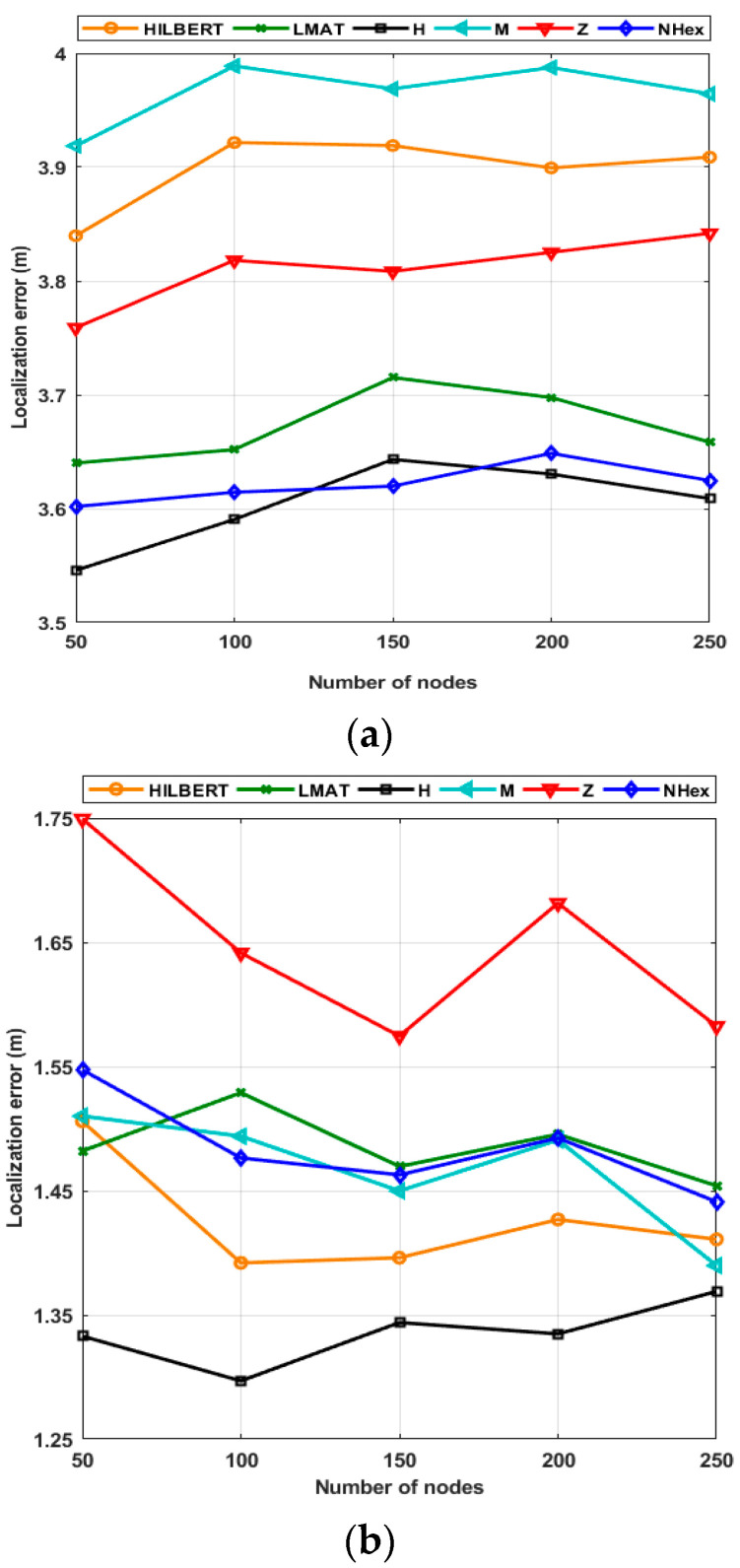
Scalability of the path planning models in terms of localization error performance (**a**) WCL, and (**b**) APT.

Scalability means localization performance is independent of the number of UNNs in the ROI. In this paper, the number of UNNs has been changed from 50 to 250 and uses the resolution of 1 and a std of noise *σ* = 7 to test the path planning models’ scalability. Figure 17a shows the change of the path planning models’ average error performance according to different node volume for the WCL method. First of all, it can be seen from Figure 17a that the H-curve and the proposed NHexCurves models show the best error performance of all the node numbers. Additionally, it can be said that the localization error values of NHexCurves are almost unchanged and remain constant against the changing number of nodes. When the scalability performance of the models for WCL is evaluated, it is observed that the error performance of all models does not change much with the changing volume in nodes. This can be explained by the fact that the localization method used is WCL and the statistical distribution of UNNs deployed on the network is uniform. In terms of localization error performance, it can be said that all mobile path planning models are largely scalable for the WCL method. If the same evaluation is applied to the APT localization method, it cannot be said that the error performance of all models varies significantly with the changing number of nodes again. The error values are lower due to the nature of the APT method, and again, the H-curve showed the best error performance in all node count cases. Although the models’ error values are similar, the Z-curve is set apart from the other models because it produces a slightly higher error value. The error value of the proposed model NHexCurves changed much less than other models with the changing volume of nodes. Therefore, it can be said that NHexCurves is the best model in terms of scalability performance compared to other models for both WCL and APT localization methods.

**Figure 18:**
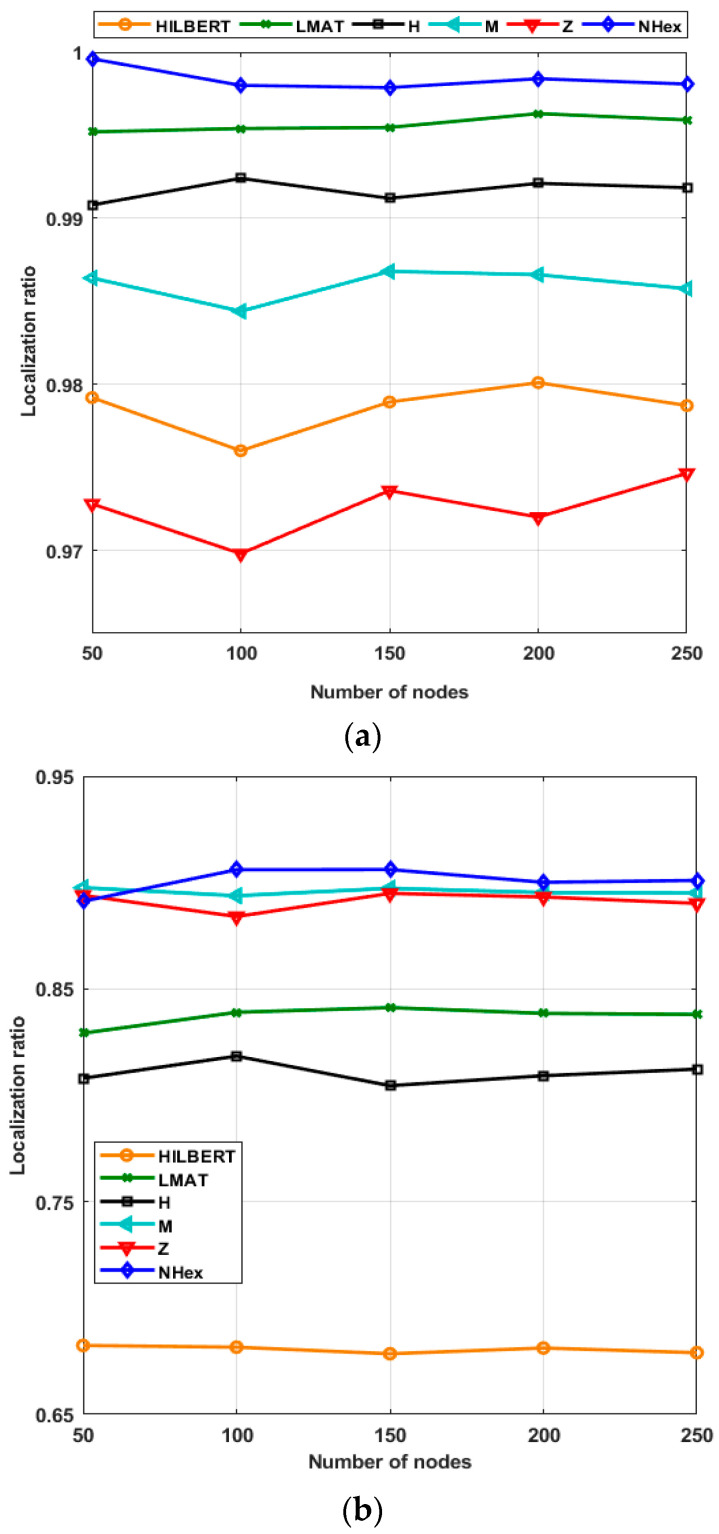
Scalability of the path planning models in terms of localization ratio performance (**a**) WCL, and (**b**) APT.

Figure 18a shows the localization ratio performances of the path planning schemes for the WCL according to the varying number of nodes. As seen from the figure, each path planning model has localized almost all of its nodes in this resolution value. Moreover, the localization ratios of each method have not changed with the changing number of nodes. The same determination can be made for the APT method; the same scenario for APT appears in Figure 18b. Using this method, NHexCurves has the best localization ratio for all node numbers among path planning models. This ratio is approximately 90% and has not changed much according to the number of nodes. M-curves and Z-curve models produced results very close to NHexCurves. When considering the changing volume of nodes, the localization ratio performance of these three models is better than the other models. However, according to the changing number of nodes, the HILBERT model’s localization ratio performance was noticeably worse than the other models, which is an expected result as explained in the previous chapters.

### 5.5. Path length

The path length is the length of the distance traveled by the MA, following the proposed model as it travels through the network. Path length does not affect localization error, but it helps identify parameters such as time and energy spent for the entire localization process. Mathematical equations of path length designs are calculated according to two variables: network size, S, and the distance between both points, dx. The following equations show the mathematical expressions of each static path planning model for the obstacle-free scenario. Equations (13)–(18) show the path length of HILBERT, LMAT, Z-curve, M-curves, H-curve, and NHexCurves path planning models, respectively.

For example, if dx = 12.5 m, S = 64 (dx)^2^ is obtained. However, as can be shown from Figure 4 and Figure 5, dx is not always 12.5 m in all models.

(13) $$L_{\mathrm{Hilbert}}=\frac{S}{\mathrm{dx}}$$

(14) $$L_{\mathrm{LMAT}}=\frac{S}{\mathrm{dx}}+20\mathrm{dx}$$

(15) $$L_{Z}=\left[\left(\frac{5}{8}\times 4^{3}\right)-1\right]\mathrm{dx}+\left[\left(\frac{3}{8}\times 4^{3}\right)\right]\times \sqrt{2}\mathrm{dx}$$

(16) $$L_{M}=\frac{109}{2}\mathrm{dx}+16\sqrt{2}\mathrm{dx}$$

(17) $$L_{H}=\frac{S}{\mathrm{dx}}+18\mathrm{dx}$$

(18) $$L_{\mathrm{NHex}}=\frac{S}{\mathrm{dx}\sqrt{2}}+38\mathrm{dx}$$

**Figure 19:**
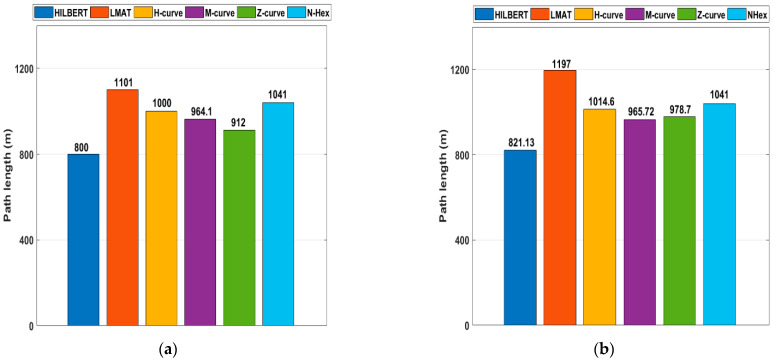
The path length for the different mobility models (**a**) obstacle-free (**b**) obstacle presence.

Figure 19a shows the path lengths of the path planning models for the obstacle-free scenario. The HILBERT model is the model that travels the shortest distance with 800 m. The Z-Curves takes a little longer, with a travel distance of about 912 m. LMAT, H-curve, NHexCurves travel longer distances than other models. However, according to most evaluation criteria, these models give better results than other models. Figure 19b shows the path lengths of the path planning models in the case of obstacle-presence. When the two figures are compared, path length differences can be seen for path planning models between the obstacle-free and obstacle-presence scenarios. These results show that when the path length and the other performance criteria are evaluated together, H-curve and NHexCurves are optimum models that can be used in the obstacle-presence scenarios.

## 6. Conclusions and Future Work

In this study, a robust path planning scheme called NHexCurves is proposed for MANAL. The proposed model guarantees that it will receive messages from at least three collinear signals to find all UNNs in the network. Therefore, the proposed path provides full coverage and high localization accuracy. The localization performance of the proposed model is compared with the performance of five known and strong static path planning models in the literature in the obstacle-presence scenario. Except for the Z-curve [9], none of these path planning models have been tested in the obstacle-presence scenario. In this study, the obstacle-presence scenario in [9] is adopted to the other four path planning models. In this study, the localization performances of all models were compared for the first time in a regular-shaped obstacle scenario according to different evaluation criteria in the same environment. With the obstacle-handling trajectories used for the models, the negative effect of the obstacle to localization was tried to be reduced. Performance evaluation criteria examined in this study for path planning models can be briefly listed as localization error depending on different parameters, localization ratio by resolution, and scalability. In this context, the simulation results made in this study can be summarized as follows:

- Generally, for the WCL method, NHexcurves, and Hcurve models stand out compared to other models. For this localization method, the HILBERT model generally shows the worst performance.
- In general, for the APT method, the H-curve model is the best performing model, but the proposed NHexCurves model is superior to other models, especially at low resolutions. For this localization method, the Z-curve model has generally been the worst performing model.
- For the APT method, the localization performance of the LMAT model was obtained poorly at low resolutions. However, the localization performance of the same model at low resolutions in WCL was achieved very well. This shows that a path planning model that performs well in one localization method may not perform well in another method.
- It can be said that the model proposed for both localization methods and all performance evaluation criteria stands out compared to other models at low resolutions. The condition of the proposed model in other conditions is also very good. Therefore, NHexCurves is an effective static path planning model that can be used in WSNs in environments with regular-shaped obstacles.

Our future research topics in MANAL will likely be as follows:

- There is no investigation regarding the energy consumption or efficiency of the path planning model proposed in this study. One of our future works will be to calculate the energy consumption of the proposed model and plan a transmission power adjustment scheme to improve energy efficiency.
- In this study, the proposed model and other models are tested in the area with regular-shaped obstacles. In future work, it is planned to observe the performance of these models in areas with irregular obstacles.
- Using only one anchor, MANAL algorithms can take a long time to find all UNNs in an ROI, especially for large-scale WSNs. Therefore, the collaborative MANAL algorithm using several MAs can be designed to reduce localization time and improve localization accuracy.
- In this study, it was assumed that MA did not go through UNNs. In future studies, the problem of collision of nodes will be examined, and in the presence of this problem, localization performances of path planning models will be compared. Improvement of localization performances in the case of collision will be tried with the existing solutions proposed in the literature to solve this problem.
- Bluetooth 5.1 is a new technology aiming to improve indoor localization with techniques such as AoD and AoA [39]. Likewise, UWB technology is also very suitable for indoor applications [40]. It is planned to adopt the static path-planning logic proposed in the future study to these technologies and compare these technologies’ indoor localization performances.

## Figures and Tables

**Table 1:** Simulation parameters.

Parameters	Symbol	Value	Unit
Network size	*S*	100 × 100	m^2^
Number of UNNs	*N*	250	-
Number of MAs	*M*	1	-
Resolutions	*R*	0.5, 0.75, 1, 1.5, 2, 2.5	-
Path loss exponent	γ	3.3	-
Std. dev. of noise	σ	3, 5, 7, 9	-
Power loss at $d_{0}$	$P_{L}{(d}_{0})$	55	dB
Reference point	$d_{0}$	1	m
Transmission power	$P_{trans}$	−20 < *P_trans_* < 10	dBm
Simulation run	-	50	-

**Table 2:** Statistical characterization of all mobile path planning models in terms of localization error for WCL.

Stat. Char.	Hilbert	LMAT	H	M	Z	NHex
mean	3.9088	3.6584	* 3.6089	3.9644	3.8419	3.6247
median	3.9287	3.6673	* 3.6010	3.9704	3.8347	3.6406
std	0.1358	0.1217	0.1205	* 0.1144	0.1249	0.1224
var	0.0184	0.0148	0.0145	* 0.0131	0.0156	0.0150
min error	3.5992	* 3.3378	3.3948	3.6854	3.6056	3.3737
max error	4.2907	3.8806	* 3.8559	4.2104	4.2000	3.8576

* is the best value.

**Table 3:** Statistical characterization of all mobile path planning models in terms of localization error for APT.

Stat. Char.	Hilbert	LMAT	H	M	Z	NHex
mean	1.4268	1.4954	* 1.3347	1.4909	1.6815	1.4925
median	1.4077	1.5029	* 1.3214	1.4644	1.6637	1.4644
std	0.1784	0.1611	* 0.1493	0.1889	0.1767	0.1738
var	0.0318	0.0260	* 0.0223	0.0357	0.0312	0.0302
min error	* 0.9783	1.0489	1.0091	1.0956	1.2005	1.1397
max error	1.9102	1.8845	* 1.6325	1.8920	2.0494	1.8749

* is the best value.

**Table 4:** Statistical characterization of all mobile path planning models in terms of std of errors for WCL.

Stat. Char.	Hilbert	LMAT	H	M	Z	NHex
mean	1.4833	1.7980	* 1.3285	1.7454	1.6763	1.4033
median	1.3310	1.6781	* 1.1280	1.5824	1.5449	1.2417
std	1.1419	1.2291	1.0783	1.2312	1.2643	* 0.9193
var	1.3040	1.5107	1.1628	1.5160	1.5983	* 0.8451
min error	0.0200	0.1179	0.0568	0.0278	0.0319	* 0.0159
max error	* 4.0808	6.2841	6.1966	5.5430	5.4294	4.54820

* is the best value.

**Table 5:** Statistical characterization of all mobile path planning models in terms of std of errors for APT.

Stat. Char.	Hilbert	LMAT	H	M	Z	NHex
mean	2.4015	2.3577	* 2.1395	2.4149	2.7178	2.4276
median	2.3682	2.3947	* 2.1464	2.4320	2.7006	2.4077
std	0.2354	0.1952	* 0.1581	0.2004	0.1731	0.2253
var	0.0554	0.0381	* 0.0250	0.0402	0.0300	0.0508
min error	1.8602	1.8295	* 1.8277	1.9280	2.3323	2.0347
max error	2.9942	2.7463	* 2.5091	2.8336	3.1216	2.8790

* is the best value.

## Data Availability

Not applicable.

## References

[1] Akyildiz I.F., Vuran M.C. (2010). WSN applications. Wireless Sensor Networks.

[2] Chandrasekhar V., Seah W.K., Choo Y.S., Ee H.V. (2006). Localization in underwater sensor networks: Survey and challenges. Proceedings of the 1st ACM International Workshop on Underwater Networks (WUWNet’06).

[3] Patwari N., Ash J.N., Kyperountas S., Hero A.O., Moses R.L., Correal N.S. (2005). Locating the nodes: Cooperative localization in wireless sensor networks. IEEE Signal Process. Mag..

[4] Li Z., Trappe W., Zhang Y., Nath B. Robust statistical methods for securing wireless localization in sensor networks. Proceedings of the IEEE Fourth International Symposium on Information Processing in Sensor Networks (IPSN 2005).

[5] Yildiz D., Karagol S., Tadiparthi S., Ozgonenel O., Bikdash M. A Novel Self Localization Approach for Sensors. Proceedings of the Sensor Signal Processing for Defence (SSPD).

[6] He T., Huang C., Blum B.M., Stankovic J.A., Abdelzaher T. (2003). Range-free localization schemes for large scale sensor networks. Proceedings of the 9th Annual International Conference on Mobile Computing and Networking (MobiCom’ 03).

[7] Hu Z., Gu D., Song Z., Li H. Localization in wireless sensor networks using a mobile anchor node. Proceedings of the IEEE/ASME International Conference on Advanced Intelligent Mechatronics.

[8] Han G., Jiang J., Zhang C., Duong T.Q., Guizani M., Karagiannidis G.K. (2016). A survey on mobile anchor node assisted localization in wireless sensor networks. IEEE Commun. Surv. Tutor..

[9] Rezazadeh J., Moradi M., Ismail A.S., Dutkiewicz E. (2014). Superior path planning mechanism for mobile beacon-assisted localization in wireless sensor networks. IEEE Sens. J..

[10] Kannadasan K., Edla D.R., Kongara M.C., Kuppili V. (2020). M-Curves path planning model for mobile anchor node and localization of sensor nodes using Dolphin Swarm Algorithm. Wirel. Netw..

[11] Alomari A., Comeau F., Phillips W., Aslam N. (2018). New path planning model for mobile anchor-assisted localization in wireless sensor networks. Wirel. Netw..

[12] Yildiz D., Karagol S., Ozgonenel O., Tadiparthi S., Bikdash M. Three-Dimensional Sensor Localization Using Modified 3N Algorithm. Proceedings of the IEEE 30th International Conference on Advanced Information Networking and Applications (AINA).

[13] Shen G., Zetik R., Thoma R.S. Performance comparison of TOA and TDOA based location estimation algorithms in LOS environment. Proceedings of the IEEE 5th Workshop on Positioning, Navigation and Communication.

[14] Gu S., Yue Y., Maple C., Wu C., Liu B. Challenges in mobile localization in wireless sensor networks for disaster scenarios. Proceedings of the IEEE International Conference on Automation and Computing (ICAC).

[15] Akbaş M.İ., Kantarcı M.E., Turgut D. (2015). Localization for wireless sensor and actor networks with meandering mobility. IEEE Trans. Comput..

[16] Rashid H., Turuk A.K. (2015). Dead reckoning localisation technique for mobile wireless sensor networks. IET Wirel. Sens. Syst..

[17] Koutsonikolas D., Das S.M., Hu Y.C. (2007). Path planning of mobile landmarks for localization in wireless sensor networks. Comput. Commun..

[18] Han G., Zhang C., Lloret J., Shu L., Rodrigues J.J.P.C. (2014). A mobile anchor assisted localization algorithm based on regular hexagon in wireless sensor networks. Sci. World J..

[19] Rezazadeh J., Moradi M., Ismail A.S., Dutkiewicz E. (2015). Impact of static trajectories on localization in wireless sensor networks. Wirel. Netw..

[20] Johnson D.B., Maltz D.A., Imielinski T., Korth H.F. (1996). Dynamic source routing in ad hoc wireless networks. Mobile Computing.

[21] Han G., Chao J., Zhang C., Shu L., Li Q. (2014). The impacts of mobility models on DV-hop based localization in mobile wireless sensor networks. J. Netw. Comput. Appl..

[22] Li S., Kong X., Lowe D. Dynamic path determination of mobile beacons employing reinforcement learning for wireless sensor localization. Proceedings of the IEEE 26th International Conference on Advanced Information Networking and Applications Workshops (WAINA).

[23] Li H., Wang J., Li X., Ma H. Real-time path planning of mobile anchor node in localization for wireless sensor networks. Proceedings of the IEEE International Conference on Information and Automation (ICINFA).

[24] Li X., Mitton N., Simplot-Ryl I., Simplot-Ryl D. Mobile-beacon assisted sensor localization with dynamic beacon mobility scheduling. Proceedings of the IEEE Eighth International Conference on Mobile Ad-Hoc and Sensor Systems (MASS).

[25] Wu Q., Chen Z., Wang L., Lin H., Jiang Z., Li S., Chen D. (2020). Real-time dynamic path planning of mobile robots: A novel hybrid heuristic optimization algorithm. Sensors.

[26] Chen D., Li S., Wu Q. (2021). A novel supertwisting zeroing neural network with application to mobile robot manipulators. IEEE Trans. Neural Netw. Learn. Syst..

[27] Peng Z., Wang J., Han Q.L. (2019). Path-following control of autonomous underwater vehicles subject to velocity and input constraints via neurodynamic optimization. IEEE Trans. Ind. Electron..

[28] Hu X., Chen L., Tang B., Cao D., He H. (2018). Dynamic path planning for autonomous driving on various roads with avoidance of static and moving obstacles. Mech. Syst. Signal Process..

[29] Han G., Xu H., Jiang J., Shu L., Hara T., Nishio S. (2013). Path planning using a mobile anchor node based on trilateration in wireless sensor networks. Wirel. Commun. Mob. Comput..

[30] Magadevi N., Kumar V.J.S. (2019). Energy efficient, obstacle avoidance path planning trajectory for localization in wireless sensor network. Cluster Comput..

[31] Han G., Yang X., Liu L., Zhang W., Guizani M. (2020). A disaster management-oriented path planning for mobile anchor node-based localization in wireless sensor networks. IEEE Trans. Emerg. Top. Comput..

[32] Blumenthal J., Grossmann R., Golatowski F., Timmermann D. Weighted centroid localization in zigbee-based sensor networks. Proceedings of the IEEE International Symposium on Intelligent Signal Processing (WISP).

[33] Savvides A., Han C.C., Strivastava M.B. (2001). Dynamic fine-grained localization in ad-hoc networks of sensors. Proceedings of the 7th Annual International Conference on Mobile Computing and Networking (MobiCom’01).

[34] Ou C.H., He W.L. (2013). Path planning algorithm for mobile anchor-based localization in wireless sensor networks. IEEE Sens. J..

[35] Zamalloa M.Z., Krishnamachari B. (2007). An analysis of unreliability and asymmetry in low-power wireless links. ACM Trans. Sens. Netw..

[36] Dezfouli B., Radi M., Razak S.A., Pink T.H., Abu Bakar K. (2015). Modeling low-power wireless communications. J. Netw. Comput. Appl..

[37] Chipcon, Dallas, TX, USA, CC1000 Low Power Radio Transceiver. https://www.ti.com/lit/ds/symlink/cc1000.pdf?ts=1613935469730&ref_url=https%253A%252F%252Fwww.ti.com%252Fproduct%252FCC1000.

[38] Rappaport T.S. (1996). Wireless Communications: Principles and Practice.

[39] Cominelli M., Patras P., Gringoli F. (2019). Dead on arrival: An empirical study of the Bluetooth 5.1 positioning system. Proceedings of the 13th International Workshop on Wireless Network Testbeds, Experimental Evaluation & Characterization (WINTECH’19).

[40] Alarifi A., Al-Salman A., Alsaleh M., Alnafessah A., Al-Hadhrami S., Al-Ammar M.A., Al-Khalifa H.S. (2016). Ultra wideband indoor positioning technologies: Analysis and recent advances. Sensors.

